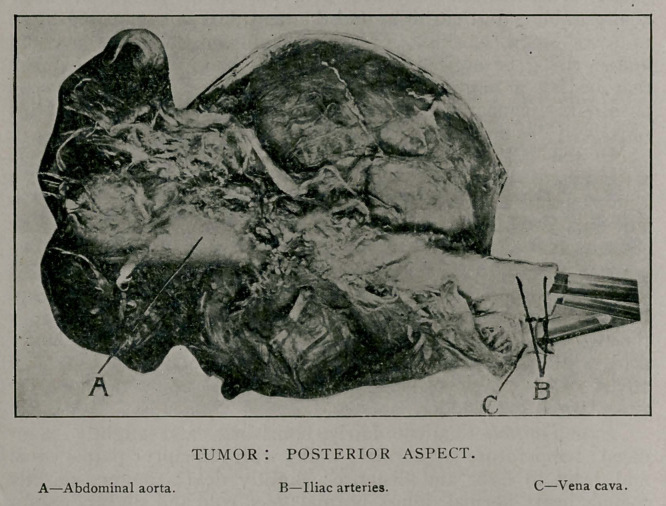# Corrected Report of an Abdominal Aneurism

**Published:** 1904-08

**Authors:** Marshall Clinton

**Affiliations:** 466 Franklin Street, Buffalo, N. Y.; Attending Surgeon, Sisters of Charity, and Erie County Hospitals, Buffalo, N. Y.; Instructor in Surgery in the University of Buffalo


### Corrected Report of an Abdominal Aneurism.

By MARSHALL CLINTON, M. D., Buffalo, N. Y.,

Attending Surgeon, Sisters of Charity, and Erie County Hospitals, Buffalo, N. Y.; Instructor in Surgery in the University of Buffalo.

In The Buffalo Medical Journal, of September, 1901, the author reported "Preliminary Notes on a Case of Abdominal Aneurism." Since that publication developments in the pathological findings render it necessary to correct the report of the case. The interesting clinical history, the operative feature and the postmortem findings make a valuable record. The history of the patient is as follows:

W. H. G., aged 57; U. S.; carpenter; married. Admitted Erie County Hospital, July 8, 1901.

*Family History.*—Father died at the age of 65 of cancer of the face; mother died of congestion of lungs. Two brothers and five sisters. One sister has female trouble, one died of consumption, one of heart disease, the rest all well and healthy. Has two children, both boys—one 19 years old at Craig Colony for Epileptics, the other in normal health.

*Previous History.*—Never has used alcohol nor tobacco, has always worked hard at his trade, doing some painting occasionally. Had measles and whooping cough when a child. Thirty years ago used to have attacks of vomiting at night after eating heavily. Four or five years ago had frequent attacks of jaundice which would be relieved by doses of Rochelle salts. Had dizzy spells frequently, fifteen years ago, and headaches at night. Two years ago was bothered with palpitation on exertion.

*Present Illness.*—Four years ago was lifting a heavy piece of lumber when he felt something give way in his abdomen. Had considerable pain that night and the next day, but managed to go to work again, working for six or eight weeks. One morning before rising he noticed as he passed his hand over his abdomen that he felt a lump on the right side. After he stood up and passed his urine the lump disappeared, so he put it out of his mind and did not notice it until in bed some nights later.

In the fall of 1900, was walking three miles to his home after a hard day's work, when he felt something give out in his abdomen and a severe pain followed. He sat down by the roadside and rested for fifteen minutes and the pain disappeared. As soon as he began to try to walk the pain would come back, so he was forced to take short walks and long rests, and in this way reached home. The pain stopped when he went to bed, and he got up the next morning apparently all right. Did light work for two weeks, when the pain returned and became a dull heavy ache while on his feet, disappearing when he was in bed. On advice of his physician he came to Buffalo to be operated on for an enlarged gallbladder.

*Examination.*—Patient fairly nourished, and slightly jaundiced; temperature subnormal; walks with difficulty; pupils equal and react to light and distance; slightly deaf; tongue, slightly coated; heart, normal; lungs, normal; feet, cold and slightly numb; pulse, 74; respiration, normal; abdomen shows a bulging mass in the right hypochondriac region, dome-shaped, four by two inches wide and cystic in feel. No pulsation can be seen or felt and no thrill or bruit can be heard over the mass or over the femorals.

*Diagnosis.*—Stenosis of the cystic duct and retention cyst of the gallbladder.

*Operation,* July 25, 1901.—An incision, four inches long, parallel with the border of the ribs and over the prominent part of the tumor down to the peritoneum revealed a dark almost black tumor adherent to the peritoneum. As the color was so unlike a gallbladder an aspirating needle was plunged into the center of the mass and was followed by a gush of bright, red blood through the lumen of the needle. Adhesions along the lower border were separated to permit of better inspection and after the lower edge was freed, it was seen to have a distinct fibrous capsule and to pulsate. The needle introduced an inch and a half from the first point of entrance was followed by blood, as in the first trial. A patient effort was made to introduce silver wire through the needle, but the available wire was unsuitable in size and would kink as soon as the end struck the opposite side of the sac. The openings made by the needle were closed by holding the finger lightly over them for a few minutes, when a clot would form under the finger and the hole be plugged. It was possible to introduce the finger along the lower border of the sac in the direction of the abdominal aorta.

TUMOR: POSTERIOR ASPECT.

A—Abdominal aorta. B—Iliac arteries. C—Vena cava.

At two different times the patient was anesthetised locally with cocaine and the tumor exposed at its upper margin. A small trocar previously insulated with shellac was carefully introduced into the body of the tumor and was followed each time by a gush of bright red blood through the needle. Silver wire was passed for a few inches beyond the end of the needle, but it kinked and only a small amount of wire could be introduced. A weak current from a galvanic battery with a low amperage was applied for an hour, and two weeks later a second application was made for half an hour.

Following these procedures the patient seemed to improve markedly as far as sensory symptoms went, but there was no improvement in his loss of motor centers. His pain and discomfort left him for several months and the pulsations in the tumor were apparently lessened. The size of the tumor from the time it was treated by the galvanic current did not increase in size nor did the patient have any further pain referable to the tumor. He was suffering from a chronic cystitis and died January 28, 1903, from uremia.

By permission of his relatives we were able to remove the tumor through the operation wound. The mass was found to have separated the abdominal aorta and the vena cava by growing up between them and had given the vena cava a sharp curve. From the size, shape, and contour of the tumor no suspicion was aroused that we were dealing with anything but an aneurism of the abdominal aorta.

When a section of the entire tumor was made a solid mass was found which had no communication with the lumen of the aorta other than by small vessels and the appearance was that of a cancerous mass. The central portion was made up of a mass of debris lined by a cyst wall that had evidently been filled with a mass of blood under pressure, which accounted for the flow of bright blood whenever a needle was introduced. Clinically, the growth was a tumor of mixed type, of the cystosarcoma variety, while sections pronounced it to be a perithelioma.

The case is interesting as illustrating what an ordinary lymph gland in the retroperitoneal space may degenerate or develop into, and how a growth technically impossible to remove may be retarded by various means.

466 Franklin Street.

## Figures and Tables

**Figure f1:**